# The innate immune receptor Nlrp12 suppresses autoimmunity to the retina

**DOI:** 10.1186/s12974-022-02425-x

**Published:** 2022-03-21

**Authors:** Ellen J. Lee, Ruth J. Napier, Emily E. Vance, Sydney J. Lashley, Agnieszka D. Truax, Jenny P. Ting, Holly L. Rosenzweig

**Affiliations:** 1 VA Portland Health Care System, Portland, OR USA; 2 Dept. of Molecular Microbiology & Immunology, Oregon Health & Science University, Portland, OR USA; 3 Lineberger Comprehensive Cancer Center, University North Carolina, Chapel Hill, NC USA; 4 Oregon Health & Science University, VA Portland Health Care System, 3710 SW US Veterans Hospital Rd., Bldg 103, Room E-222, Mail stop: VA R&D-14, Portland, OR 97239 USA; 5 Lineberger Comprehensive Cancer Center, Depts. Genetics and Microbiology-Immunology, University of North Carolina, Chapel Hill, NC USA

**Keywords:** Nlrp12, Pattern recognition receptor, Nod-like receptor, Uveitis

## Abstract

**Background:**

Nod-like receptors (NLRs) are critical to innate immune activation and induction of adaptive T cell responses. Yet, their role in autoinflammatory diseases of the central nervous system (CNS) remains incompletely defined. The NLR, Nlrp12, has been reported to both inhibit and promote neuroinflammation in an animal model of multiple sclerosis (experimental autoimmune encephalomyelitis, EAE), where its T cell-specific role has been investigated. Uveitis resulting from autoimmunity of the neuroretina, an extension of the CNS, involves a breach in immune privilege and entry of T cells into the eye. Here, we examined the contribution of Nlrp12 in a T cell-mediated model of uveitis, experimental autoimmune uveitis (EAU).

**Methods:**

Mice were immunized with interphotoreceptor retinoid-binding protein peptide 1–20 (IRBP_1–20_) emulsified in Complete Freund’s adjuvant, CFA. Uveitis was evaluated by clinical and histopathological scoring, and comparisons were made in WT vs. Nlrp12^−/−^ mice, lymphopenic Rag1^−/−^ mice reconstituted with WT vs. Nlrp12^−/−^ CD4^+^ T cells, or among bone marrow (BM) chimeric mice. Antigen-specific Th-effector responses were evaluated by ELISA and intracellular cytokine staining. Cellular composition of uveitic eyes from WT or Nlrp12^−/−^ mice was compared using flow cytometry. Expression of Nlrp12 and of cytokines/chemokines within the neuroretina was evaluated by immunoblotting and quantitative PCR.

**Results:**

Nlrp12^−/−^ mice developed exacerbated uveitis characterized by extensive vasculitis, chorioretinal infiltrates and photoreceptor damage. Nlrp12 was dispensable for T cell priming and differentiation of peripheral Th1 or Th17 cells, and uveitis in immunodeficient mice reconstituted with either Nlrp12^−/−^ or WT T cells was similar. Collectively, this ruled out T cells as the source of Nlrp12-mediated protection to EAU. Uveitic Nlrp12^−/−^ eyes had more pronounced myeloid cell accumulation than uveitic WT eyes. Transplantation of Nlrp12^−/−^ BM resulted in increased susceptibility to EAU regardless of host genotype, but interestingly, a non-hematopoietic origin for Nlrp12 function was also observed. Indeed, Nlrp12 was found to be constitutively expressed in the neuroretina, where it suppressed chemokine/cytokine induction.

**Conclusions:**

Our data identify a combinatorial role for Nlrp12 in dampening autoimmunity of the neuroretina. These findings could provide a pathway for development of therapies for uveitis and potentially other autoinflammatory/autoimmune diseases of the CNS.

**Supplementary Information:**

The online version contains supplementary material available at 10.1186/s12974-022-02425-x.

## Background

Uveitis, or intraocular inflammatory disease, accounts for a significant proportion of blindness in the US [1, 2]. Autoimmune responses that overcome the protective mechanisms of ocular immune privilege constitute one of a number of possible etiologies of uveitis. Ocular immune privilege is normally maintained by physical sequestration of ocular antigens such as interphotoreceptor retinoid-binding protein (IRBP) from the systemic immune system by the blood-retinal barrier, active immunosuppressive microenvironment of the eye [3], and peripheral tolerance mechanisms. Together, these mechanisms suppress immune/inflammatory processes that would otherwise destroy the terminally differentiated photoreceptors of the neuroretina that are critical for vision [4]. Uveitis can occur as an isolated entity or in association with systemic autoimmune diseases [5, 6], including multiple sclerosis (MS) [7–9]. Many clinical and histopathological aspects of uveitis are mimicked in an animal model, experimental autoimmune uveitis (EAU), which is induced by immunization with retinal proteins such as IRBP [4, 10]. To date, discoveries of adaptive immune mechanisms in uveitis using this model have led to therapies that target T cells, such as CD4^+^ effector T cells polarized to the Th17 phenotype, a driving pathogenic mechanism of EAU [11].

Innate immune sensors such as Nod-like receptors (NLRs) detect an array of potential pathogens and activate microbicidal and inflammatory responses required for their elimination [12]. NLRs reside in the cytosol where their activation leads to their oligomerization, formation of large molecular scaffolds, signal transduction, and subsequent restoration of cellular homeostasis [13]. Interestingly, a number of NLRs are linked to human inflammatory disorders including ones accompanied by ocular manifestations such as uveitis, thereby underscoring their importance in immune homeostasis and disease. The NLR, *NLRP12* has been linked to inflammatory diseases with ocular involvement [14–16], including MS [17]. While its agonist is yet to be identified, Nlrp12 has been shown to negatively regulate inflammation through suppression of inflammatory pathways, e.g., canonical and non-canonical NF-κB signaling [18–27], although there are also contrasting reports of Nlrp12 function in promotion of inflammatory pathways [24, 28–30].

Nlrp12 has recently been implicated in regulation of neuroinflammation in the context of a murine model of MS, experimental autoimmune encephalomyelitis (EAE). Nlrp12^−/−^ mice were shown to be highly susceptible to EAE, which was attributed to an exacerbated Th1 response [31] and microglia activation [32]. Conversely, Nlrp12 has also been found to promote EAE. Nlrp12^−/−^ mice developed a milder form of EAE that was mediated by T cell-intrinsic control of cytokine production [33]. This would be consistent with another report showing that Nlrp12 promotes development of a spontaneous form of EAE in 2D2 T cell receptor (TCR) transgenic (Tg) mice and its role in suppressing TCR signaling pathways [31]. These contradictory findings not only indicate an incomplete understanding of how Nlrp12 modulates EAE, but also highlight potential counter-regulatory functions of NLRs within T cells in shaping autoinflammatory disease. Here, we sought to investigate the function of Nlrp12 using EAU, a T cell-mediated model of CNS autoimmunity similar to EAE.

Our findings demonstrated a role for Nlrp12 in downregulation of inflammation and protection against EAU. The mechanism is not regulated inherently by T cells, but may be attributed to a multi-cellular mechanism involving BM-derived myeloid cellular responses as well as local Nlrp12 function in the neuroretina. These data provide novel insight into the functioning of Nlrp12 in suppression of T cell-mediated uveitis through integration of innate immune and ocular responses.

## Methods

### Mice

C57BL/6J (stock #000664) and Rag1^−/−^ (stock #002216) mice were purchased from The Jackson Laboratory (Bar Harbor, ME, USA). Nlrp12^−/−^ mice on C57BL/6J background strain [24] were used. All mice were bred in-house under specific pathogen-free (SPF) conditions at the VA Portland Health Care System and were used between the ages of 6–10 weeks. Studies were carried out in accordance with the U.S. Department of Health and Human Services Guide for the Care and Use of Laboratory Animals and were performed under institutional protocols approved at the VA Portland Health Care System, which adhere to the ARVO Statement for the Use of Animals in Ophthalmic and Vision Research.

### Induction of EAU

Uveitis was induced as described [34] with modification. In brief, mice were immunized with 500 µg of human IRBP_1–20_ peptide (Anaspec, Fremont, CA) emulsified in Complete Freund’s Adjuvant (CFA; Sigma) and supplemented with *Mycobacterium tuberculosis* (strain H37Ra; BD Difco, Detroit, MI) to a final concentration 2.5 mg/ml. On the day of immunization, mice were also i.p. injected with 1 µg *Bordetella pertussis* toxin (PTX; List Biological Laboratories, Campbell, CA).

### Evaluation of uveitis

Clinical uveitis was evaluated by topical endoscopic fundus imaging (TEFI). Fundoscopy photos of the central and peripheral retina were scored in masked fashion using a modified 4-point grading scale based on a previously published system [35]; Grade 1: inflammation limited primarily to the optic nerve head and vasculature, and discrete lesions covering up to 50% retina; Grade 2: inflammation encompassing the optic nerve head, vasculature, and discrete lesions covering up to 75% area of retina; Grade 3: inflammation at the optic nerve head, vasculature, and coalescing retinal lesions with less than 25% visible retina; Grade 4: inflammation at the optic nerve head, vasculature, coalescing retinal lesions, and retinal detachment. For histological assessment, paraffin-embedded eyes were sectioned through the optic nerve, stained with hematoxylin and eosin (H&E), and scored in masked fashion based on a standardized 4-point grading system [34] ranging from 0 (no disease) to 4 (maximum disease) [36] based on extent of retinal cell damage, retinal detachment, vasculitis, granulomatous-like lesions, hemorrhages, perivascular exudates, and optic disc infiltration. Histological photographs were obtained from similar regions of the retina, approximately 30–60 degrees from the optic nerve head, thus representing mid-peripheral retina.

### EAU induction in reconstituted lymphopenic Rag1^−/−^ mice

CD4^+^ T cells were isolated (> 99% purity) from spleens of naïve WT or Nlrp12^−/−^ mice by negative selection (CD4^+^ Negative Selection Kit #19852, StemCell Technologies) as described [37]. CD4^+^ T cells were transferred by i.v. injection (3.5 × 10^7^ cells) into naïve Rag1^−/−^ recipients. One day later, recipients were immunized for EAU as above, and monitored for uveitis thereafter.

### Generation of bone-marrow chimeric mice

WT or Nlrp12^−/−^ recipient mice (6 wk of age) were lethally irradiated with a total of 1100 cGy (delivered in two 550 cGy doses administered 3 h apart; X-RAD 320; Precision X-ray, North Branford, CT) as established [37] and transplanted with bone-marrow cells (4 × 10^6^ cells) derived from femurs of WT or Nlrp12^−/−^ donor mice. Chimeric mice achieved > 95% donor engraftment 8 weeks after transplantation, as confirmed by congenic CD45.1/CD45.2 antigen expression, using CD45.1-expressing B6.SJL-*Ptprc*^*a*^* Pepc*^*b*^/BoyJ (stock #002014; The Jackson Laboratory) as WT donors. Chimeric mice (CD45.2 C57BL/6J) were immunized for EAU as described above at 8 week post-BM transplantation. Eyes were then evaluated 28 days later for uveitis.

### Antigen-T cell stimulation assay for ELISA and intracellular cytokine staining

Cytokine production of IRBP-responsive CD4^+^ T cells was measured as reported [34]. Splenocytes of immunized mice (20 day post-immunization) were seeded (5 × 10^5^ cells/well) in 96-well plates (Corning Inc., Corning, NY) in HL-1 medium (Lonza, Basel, Switzerland) containing gentamicin (10 μg/ml), HEPES (10 mM), sodium pyruvate (1 mM), and nonessential amino acids (BioWhittaker). Cells were stimulated with 20 µg/ml IRBP_1–20_ peptide (Anaspec) for 18 h, after which supernatants were assessed for IFNγ, IL-17A/F, IL-4, and IL-22 production by sandwich ELISA as per manufacturer’s instructions (DuoSet®, R&D). For intracellular cytokine staining, cells were stimulated as above [34] with PMA (20 ng/ml; LC Labs), ionomycin (200 ng/ml; LC Labs, Woburn, MA) and Brefeldin A (1 µg/ml; BD GolgiPlug™, BD Biosciences) during the last 4 h. Cells were blocked with mAb to FcγRIII/I (2.4G2, BD) and subsequently incubated with LIVE/DEAD™ Aqua (Life Technologies) along with mAbs to: CD45, CD4, and CD8 (BD Bioscience). Cells were then fixed and permeabilized (BD Cytofix/Cytoperm™ Fixation/Permeabilization solution Kit (BD Biosciences), and incubated with fluorescently-labeled antibodies (BD Bioscience) specific to: IL-17A (TC11-18H10, BD Pharmingen), IFNγ (XMG1.2, BD Pharmingen). Flow cytometry was performed on a BD LSRFortessa™ (BD Biosciences) and analyzed using FlowJo (Becton, Dickinson, & Company).

### In vitro autologous APC-T cell co-cultures

Autologous criss-cross cultures of naïve APCs and CD4^+^ T cells was performed as described [37]. Briefly, APCs were purified from splenocytes (of naïve mice) that had been depleted of T cells (using EasySep Positive selection kit II for CD90.2/Thy1.2 cells; StemCell Technologies) and were confirmed by flow cytometry to be > 97% pure (i.e., devoid of T cells). APCs were incubated for 2 h in round-bottom 96-well plates (1.2 × 10^6^ cells/well) prior to the addition of T cells or IRBP_1–20_ peptide (20 µg/ml). Splenic-derived CD4^+^ T cells were purified from WT or Nlrp12^−/−^ mice (immunized 14 days prior, pooled *n* = 5/genotype) by negative selection (StemCell Technologies), added to the APCs (3 × 10^5^ cells/well), and co-cultured for 24 h in the presence of IRBP_1–20_ peptide (20 µg/ml). Cytokine levels in supernatants were measured by ELISA according to manufacturer’s instruction (DuoSet®, R&D).

### Multiplex, quantitative real-time PCR (RT-qPCR)

Extraction of total RNA from isolated neuroretina was carried out using RNeasy kit (Qiagen) and cDNA was synthesized (Reverse Transcription Kit; Applied Biosystems) as described [37]. Multiplex-transcript analysis of cytokine/chemokine expression was performed using quantitative real-time PCR (RT^2^Profiler™ PCR Gene Expression Assay kit, SABiosciences) and PRISM Sequence Detection system (Applied Biosystems). Controls were performed to confirm the lack of contaminating genomic DNA. Gene expression levels were determined by standard comparative ΔΔCt method and normalized to the average of 5 housekeeping genes within the same sample (*Gusb*, *Hprt*, *Hsp90ab1*, *Gapdh*, *Actb*), all of which were expressed at similar levels. Array results are combined (5 retinas pooled/group) and are expressed as fold relative to adjuvant-injected WT animals.

### (RT-qPCR) for Nlrp12

Transcriptional analysis of *Nlrp12* was carried out as detailed [24]. Total RNA was extracted from micro-dissected ocular tissues (pooled from 6 naïve mice; RNeasy kit; Qiagen) and cDNA synthesized (Reverse Transcription Kit; Applied Biosystems). RT-qPCR for *Nlrp12*, *Gapdh* and *Actb* mRNA levels was performed using SYBR Green Supermix (Bio-Rad) and analyzed with the comparative cycle threshold method as well as comparing the range of expression of *Nlrp12* to control genes (*Gapdh*, *Actb*). Data are expressed relative to *Nlrp12* expression in spleen.

### Immunoblotting

Protein was extracted from ocular tissues (6 eyes from 3 naïve mice pooled/tissue) as described [38]. Equal amounts of protein per sample were electrophoresed on a 12% sodium dodecyl sulfate (SDS) polyacrylamide gel (Bio-Rad) and transferred onto a polyvinylidene difluoride membrane (Millipore). Membranes were incubated with primary antibodies: anti-Nlrp12 (Invivogen) and anti-β-actin (Sigma), which were detected by IRDye (680)- and IRDye (800)-conjugated secondary antibodies. Visualization and analysis of target protein expression was carried out using Odyssey Classic® and Image Studio (LI-COR Biosciences).

### Cellular composition of ocular infiltrate

Eyes from immunized mice (21 day post-immunization, 5 eyes pooled/group) were harvested and the lenses removed. Single cell suspensions of eye tissues were then prepared and stained as described [34] using fluorescently-labeled antibodies (CD45, CD3, CD4, CD8, CD11c, CD11b, B220, Gr1, F4/80, MHC-II, CD40, CD80; BD Bioscience) and fixed with 1% paraformaldehyde. Dead cells were excluded based on viability staining, so that only live cell events were collected. The LSRFortessa (BD Biosciences) and FlowJo (Becton, Dickinson & Company) were used for analysis, with compensations for spectral overlaps within each sample. The gating strategy as defined [37] was based on control samples stained with corresponding isotype control antibodies (IC Ab). Equal numbers of live events (200,000) were collected and singlets were gated from forward and side scatter plots in identical fashion for each group, after which the frequencies and numbers of cells were determined by CD45^+^ positive staining. T cells were defined as: live, CD45^+^, B220^−^, CD3^+^ and CD4^+^ or CD8^+^. For Fig. 3A–C, hematopoietic monocyte/macrophages were distinguished from microglia based on CD45 expression, with macrophages being CD45^high^, CD11c^lo^, F4/80^high^ and microglia being CD45^lo^CD11c^lo^F4/80^lo^. Neutrophils were defined as CD45^high^, CD11c^lo^, F4/80^lo^GR-1^high^.

### Statistical analysis

Data are presented as mean ± SEM. Complex data sets were compared by ANOVA with Tukey–Kramer HSD or Newman–post-hoc tests for multiple comparisons. Statistical differences between groups were determined using the Mann–Whitney *U* test for in vivo studies or the Student’s *t* test for in vitro experiments with Prism (GraphPad Software, Inc.). For the flow cytometry studies in which eyes were from groups of animals and the entire study was repeated, statistical differences were determined using an unpaired, two-tailed Student’s *t* test, which has been validated for use when comparator groups have *n* values between 2 and 5 [39]. Values of *p* < 0.05 were considered to be statistically significant.

## Results

### Nlrp12-deficiency enhances autoimmune uveitis

**Fig. 1:**
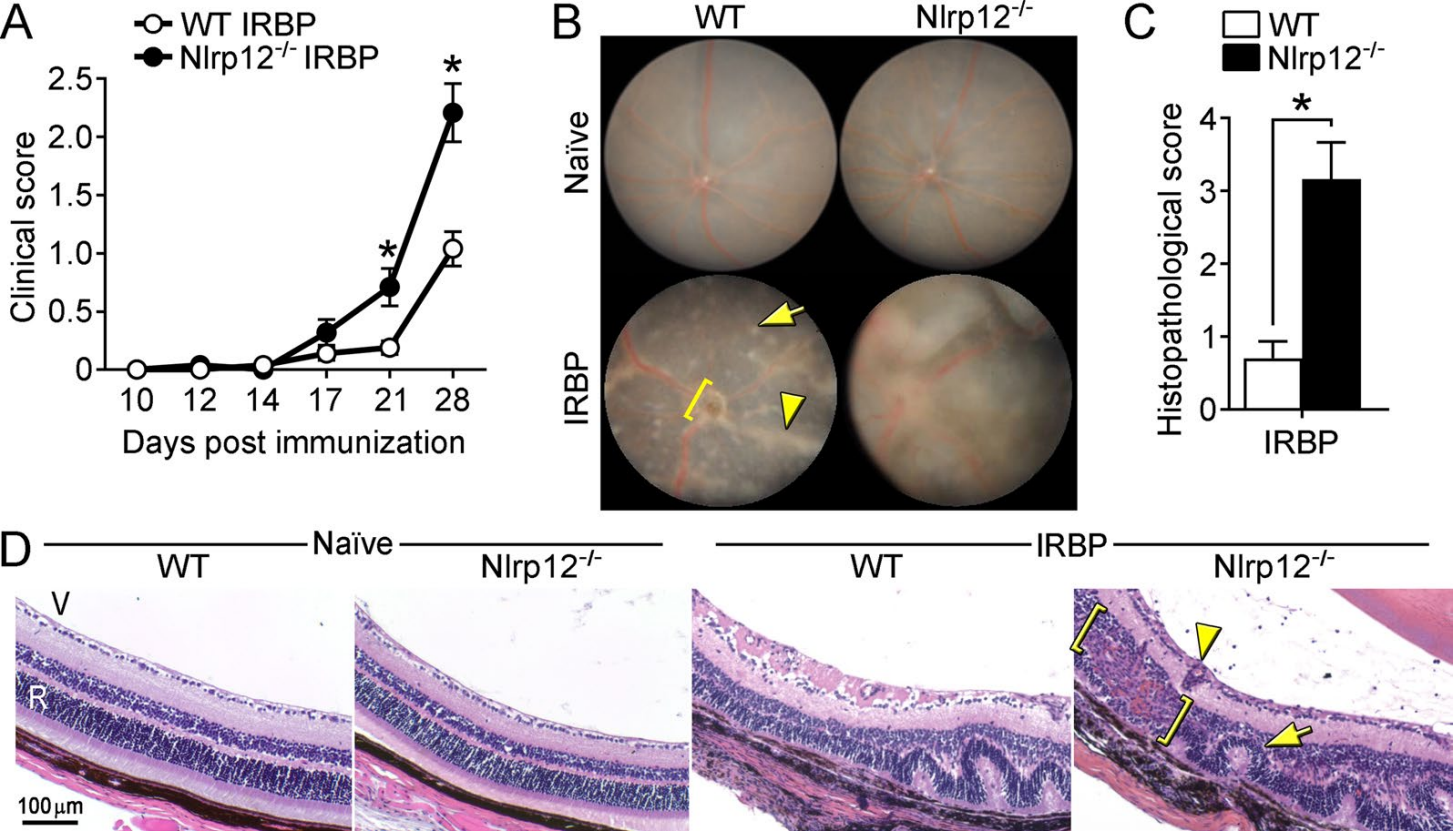
Nlrp12-deficiency exacerbates autoimmune uveitis: WT and Nlrp12^−/−^ mice were immunized with IRBP_1–20_ peptide and evaluated for uveitis. **A** Clinical scoring of eyes (*n* = 12–13 mice/group combined from 2 studies). **B** Fundoscopic images of the posterior pole of naïve or at 28 day post-immunization (WT-IRBP = grade 1; Nlrp12^−/−^-IRBP = grade 3) as compared to naive mice (WT and Nlrp12^−/−^ = grade 0). Corresponding adjuvant-immunized eyes are shown in Fig. 4B. Examples of pathological features include: vasculitis (arrowhead), retinal lesion (arrow), optic nerve head inflammation (bracket). **C** Histopathology scores of eyes 28 day post-immunization. **D** Representative photographs of H&E-stained sections of the posterior segment of the eye (approximately 30–60 degrees from the optic nerve head). Examples of pathological features include: retinal fold (arrow), granulomatous formation (bracket), vasculitis (arrowhead). V, vitreous; R, retina. For A and C, data are mean ± SEM, *p < 0.05 by Mann–Whitney

To determine whether Nlrp12 is functionally relevant to uveitis, WT and Nlrp12^−/−^ mice were immunized with IRBP_1–20_ and uveitis was evaluated. Compared to WT counterparts, Nlrp12^−/−^ mice developed more severe clinical uveitis (Fig. 1A). Exacerbated clinical manifestations of uveitis in Nlrp12^−/−^ mice included more extensive vasculitis, optic nerve head inflammation, and retinal lesions (Fig. 1B) compared to naïve eyes (Fig. 1B), or corresponding adjuvant-immunized eyes (Fig. 4B). Histopathological scoring of eyes from immunized mice corroborated the increased severity of uveitis in Nlrp12^−/−^ mice (Fig. 1C), which had more subretinal hemorrhages, perivascular and chorioretinal infiltrates (with granulomatous features), and extensive retinal damage (e.g. photoreceptor ablation and diffuse atrophy) (Fig. 1D). Naïve Nlrp12^−/−^ mice display no gross physical abnormalities, and no abnormalities in cellularity of peripheral blood, bone marrow, and lymphoid tissues [24]. Similarly, we did not observe any overt abnormalities of the external eye, and the posterior ocular segments (including optic nerve and retinal vasculature) in Nlrp12^−/−^ mice appeared comparable to WT mice (Fig. 1B, D). Together, these data reveal a somewhat unanticipated protective role for Nlrp12 in uveitis.

### Nlrp12-mediated protection against uveitis occurs independent of an inherent T cellular mechanism

**Fig. 2:**
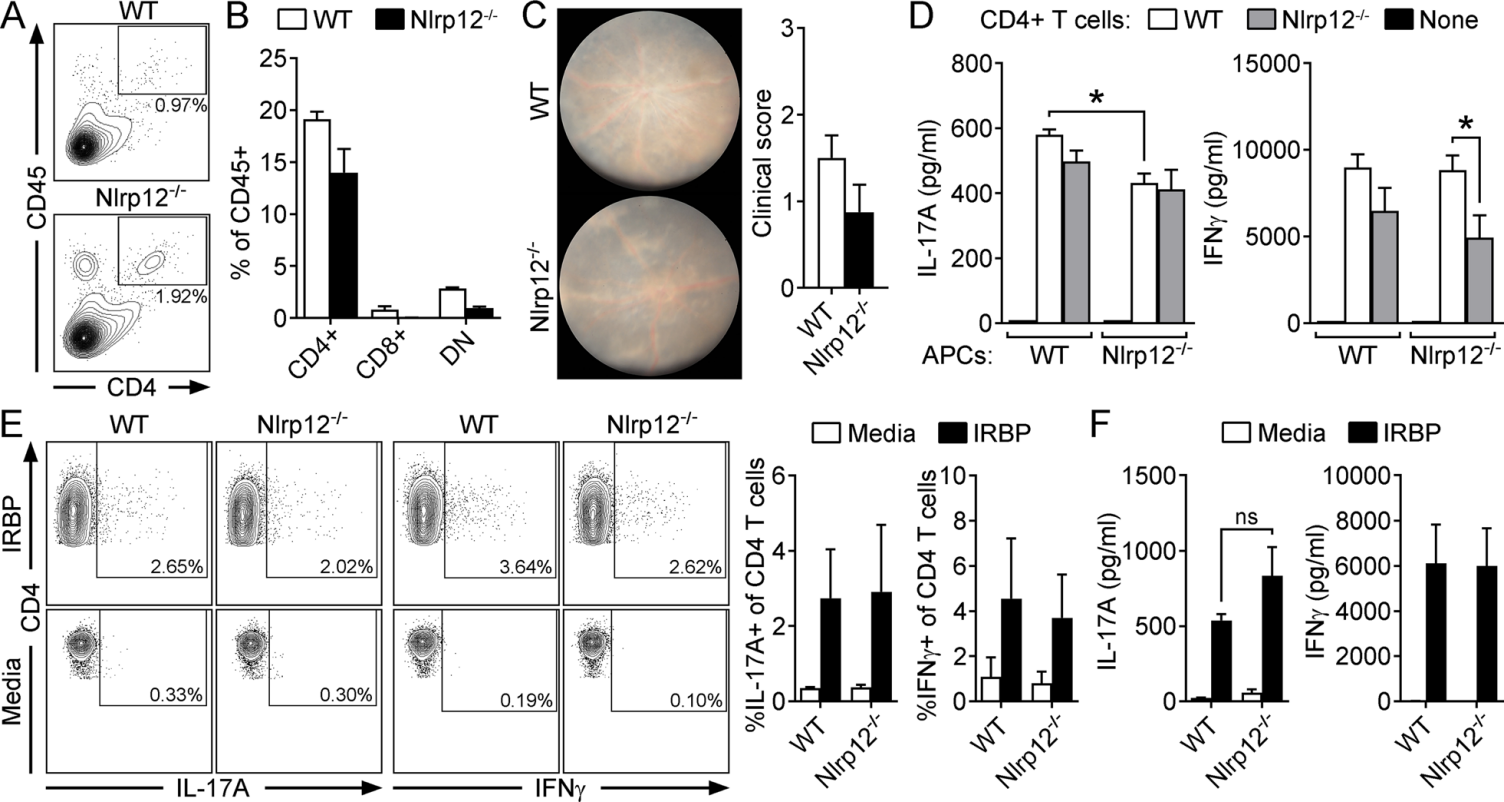
Nlrp12-mediated protection against uveitis occurs independent of an inherent T cellular mechanism. The T cell infiltrate in uveitic eyes of WT and Nlrp12^−/−^ mice was evaluated by flow cytometry 21 day post-immunization (**A**). CD4^+^ cells were identified from gated live, singles that were CD45^+^ and identified as CD4^+^ CD8^−^. Data are of pooled eyes (5 mice/group) and representative of 3 independent experiments. **B**, **C** Rag1^−/−^ mice injected with CD4^+^ T cells purified from naïve, WT or Nlrp12^−/−^ donors. The reconstituted Rag1^−/−^ mice were then immunized for EAU and evaluated for uveitis. Splenocytes of CD4^+^ T cell-reconstituted Rag1^−/−^ recipients harvested 28 day post-immunization were analyzed by flow cytometry for proportions of indicated T cell subsets (DN, double negative) (**B**). Clinical uveitis 28 day post-immunization was assessed by fundoscopy. Representative fundus photographs (**C**, left) and clinical uveitis scores are shown (**C**, right). **D** Autologous criss-cross cultures were performed to evaluate Nlrp12 function within APCs in induction of Th1/Th17 immunity. IRBP-reactive CD4^+^ T cells (purified from immunized donors) were cultured with naïve, WT or Nlrp12^−/−^ APCs, and stimulated with IRBP_1–20_ peptide. As a negative control, APCs were stimulated with IRBP_1–20_ peptide in the absence of CD4^+^ T cells. Cytokine production was measured 18 h later by ELISA. Data are combined from 3 experiments and are mean ± SEM; *p < 0.05. **E** Th-effector responses were evaluated in IRBP_1–20_ peptide-stimulated splenocyte cultures from WT vs. Nlrp12^−/−^ mice at 21 d post immunization. Representative dot plot (left) and summary statistics (right, of 3 independent experiments, each with *n* = 5 mice pooled/group) showing frequencies of IL-17A and IFNγ-producing cells of live CD4^+^ T cells. **F** Splenocytes of immunized WT vs. Nlrp12^−/−^ mice were stimulated with IRBP_1–20_ peptide and cytokine levels in culture supernatants were measured 18 h later by ELISA. Data are mean ± SEM combined from 3 experiments; in each experiment splenocytes from immunized mice were pooled (5 mice/group) and cytokine measured in triplicate wells

Nlrp12 was recently implicated as a T cell-intrinsic regulator of neuroinflammation [31, 33]. Based on our observation that CD4^+^ T cells were expanded within uveitic eyes of Nlrp12^−/−^ vs. WT mice (Fig. 2A), we evaluated whether Nlrp12 might inherently control T cells in mitigating uveitis. To do this, lymphopenic Rag1^−/−^ mice were reconstituted with CD4^+^ T cells purified from naïve WT or Nlrp12^−/−^ mice, such that all cells express Nlrp12 except for the CD4^+^ T cells. Using this model system, we have previously shown that Rag1^−/−^ mice fail to develop EAU, but become susceptible when reconstituted with CD4^+^ T cells [37]; thereby confirming the prerequisite for T cells in this model. Reconstituted Rag1^−/−^ recipients were immunized with IRBP_1–20_ and the ability of Nlrp12^−/−^ vs. WT CD4^+^ T cells to expand and trigger uveitis was evaluated. WT and Nlrp12^−/−^ CD4^+^ T cell frequencies were similar after immunization (Fig. 2B). Moreover, the severity of uveitis was not significantly different (Fig. 2C), indicating that the mechanism by which Nlrp12 suppresses EAU is not T cell-intrinsic.

Although our data do not support Nlrp12 as a T cell-intrinsic regulator of EAU, Nlrp12 is highly expressed in dendritic cells (DCs) [24]. As potent APCs, DCs are important in promoting adaptive Th-effector responses such as Th1/Th17 cellular responses, which play central roles in the pathogenesis of EAU [11]. Since the above experiment in Rag1^−/−^ mice (Fig. 2B, C) did not account for Nlrp12 function within APCs in the priming of T cells, we evaluated this process in autologous co-cultures in vitro. APCs isolated from either naïve WT or Nlrp12^−/−^ mice were co-cultured with WT or Nlrp12^−/−^ CD4^+^ T cells purified from immunized animals in the presence of IRBP_1–20_. CD4^+^ T cellular production of IL-17A or IFNγ was measured from each of the four criss-cross combinations (Fig. 2D). As controls, APCs of either genotype were cultured in the presence of IRBP_1–20_ but absence of T cells; the resulting lack of cytokine production verified the T cell-specific response to antigen as well as high purity of APC preparations (> 97%; Fig. 2D). Upon IRBP_1–20_ stimulation, we found that Nlrp12^−/−^ APCs have a potential role in antigen presentation and/or priming of T cells to promote Th17 immunity, as well as Th1 immunity in Nlrp12^−/−^ T cells, as both IL-17A and IFNγ, respectively, were reduced. In the other scenarios, cytokine production was similar, irrespective of APC or CD4^+^ T cell genotype. Since this response is in opposition to the Nlrp12-mediated phenotype of heightened severity of EAU, we would surmise that this is not the cellular mechanism by which Nlrp12 suppresses EAU. As such, we further examined the peripheral expansion of Th1/Th17 responses within global Nlrp12^−/−^ mice after immunization. IRBP-stimulated cytokine production by T cells was evaluated in splenocyte cultures by intracellular cytokine staining and flow cytometry (Fig. 2E). Frequencies of both IL-17A- and IFNγ-expressing T cells (Th17 and Th1 cells respectively), were found to be similar between WT and Nlrp12^−/−^ T cells (Fig. 2E). Moreover, the production of IL-17A and IFNγ, as measured by ELISA (Fig. 2F) was also not significantly different; although a non-significant trend of increased IL-17A production by Nlrp12^−/−^ T cells was noted. Production of IL-4 (i.e. for Th2 cells) and IL-22 (Th17-associated cytokine) was comparable between both genotypes, as measured by both ELISA and flow cytometry (data not shown), further indicating that Nlrp12 expression is dispensable for T cell-triggered cytokine production. Collectively, these data rule out any T cell-mediated mechanism related to Nlrp12-mediated protection against EAU. Regarding the potential discrepancy of results from the autologous co-cultures (Fig. 2D) and that of peptide-stimulated splenocyte cultures derived from immunized Nlrp12^−/−^ mice (Fig. 2E, F), this could relate to the nuanced differences of the experimental designs, wherein cell-specific functions may be masked in the context of global knockout mice.

### Nlrp12 suppression of uveitis involves regulation of bone-marrow (BM) derived myeloid cellular responses

**Fig. 3:**
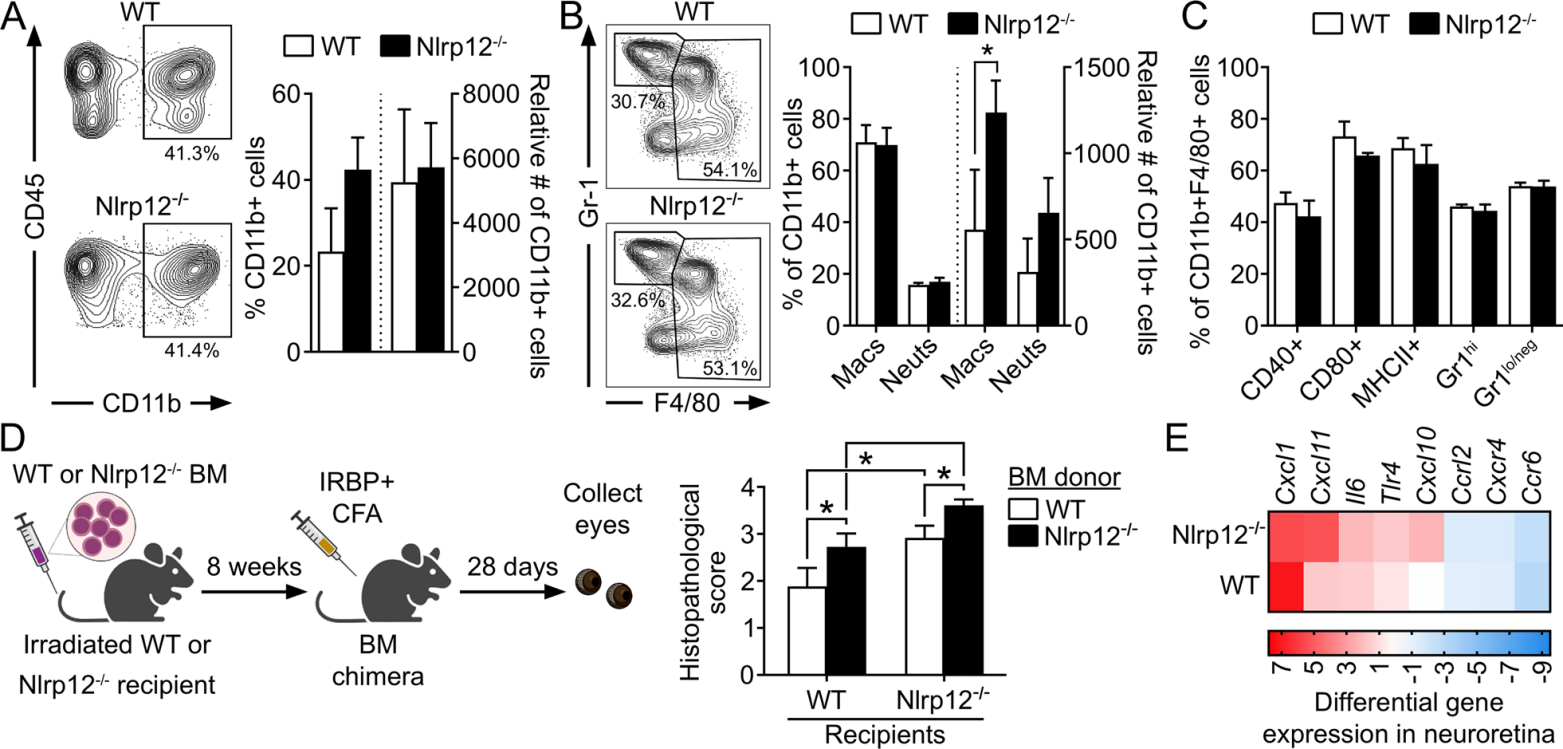
Nlrp12 suppression of uveitis involves regulation of BM-derived myeloid cellular responses. **A**–**C** Leukocytic infiltrate in the eyes of WT and Nlrp12^−/−^ mice 21 d post-immunization was evaluated by flow cytometry. Analysis was performed on live singlets expressing the pan-leukocyte marker CD45. **A** Representative contour plot and summary statistics depicting proportion and number of CD11b^+^ cells of gated CD45^+^ cells. **B** Contour plot and summary statistics of gated CD11b^+^ cells further distinguished as monocyte–macrophages (being CD45^high^, CD11c^lo^, F4/80^high^) vs. neutrophils (CD45^high^, CD11c^lo^, F4/80^lo^GR-1^high^). **C** Expression of activation markers within the gated monocyte/macrophage population. **A**–**C** Data are mean ± SEM of pooled eyes (5 mice/group) and combined across 3 independent experiments; *p < 0.05 by one-tailed Mann–Whitney. **D** Bone-marrow (BM)-chimeric mice were generated by transplantation of WT or Nlrp12^−/−^ BM into irradiated WT or Nlrp12^−/−^ recipients. Chimeras were immunized 8 weeks later, and eyes were evaluated for uveitis by histopathology 28 day post-immunization. Data are mean ± SEM (*n* = 9–10 mice/group combined from 2 experiments), *p < 0.05 by one-tailed Mann–Whitney. **E** Expression levels of cytokine/chemokine genes in neuroretinas of IRBP_1–20_-immunized WT and Nlrp12^−/−^ mice (14 day post-immunization, i.e., prior to clinical onset of uveitis) were evaluated by multiplex RT-qPCR. Colors in heatmap indicate the expression level of transcripts relative to control adjuvant-injected WT mice (*n* = 5 retinas pooled/experimental group)

Myeloid-derived cells such as macrophages are known to play a role in perpetuation of retinal damage in uveitis [40, 41] and in the eye, F4/80^+^MHCII^+^ macrophages can directly promote T cell activation via their ability to present antigen [41]. Since Nlrp12 is expressed predominately in myeloid cells [19, 24], we evaluated myeloid cell responses within eyes of immunized WT and Nlrp12^−/−^ mice. Of infiltrated hematopoietic-derived CD45^+^ leukocytes, the percentage of CD11b^+^ myeloid cells constitute ~ 20–40 percent of the cellular infiltrate (Fig. 3A), which was similar across genotypes. However, with further evaluation of gated CD11b^+^ cells, we found that the monocyte/macrophage population was significantly increased in uveitic eyes of Nlrp12^−/−^ mice (Fig. 3B) vs. neutrophils. Of the gated macrophage population both genotypes appeared phenotypically similar with regard to markers of activation (CD40, CD80), antigen-presentation function (MHCII), and inflammatory state (Gr1/Ly6G) (Fig. 3C). These data suggested a role for Nlrp12 in controlling the magnitude of bone-marrow derived monocyte/macrophages responses within the eye. Therefore, in consideration of potential mechanisms by which Nlrp12 expression is connected to myeloid-derived responses, we examined uveitis in BM-chimeric mice. After immunization, WT mice transplanted with Nlrp12^−/−^ BM cells developed more severe uveitis compared to control mice (WT mice transplanted with WT BM) (Fig. 3D). Similarly, Nlrp12^−/−^ mice transplanted with WT BM cells developed less severe uveitis compared to those transplanted with Nlrp12^−/−^ BM. Collectively, these observations support a key role for Nlrp12 in BM-derived myeloid cellular responses in mitigation of uveitis.

Furthermore, we observed an early (i.e. pre-clinical) induction of cytokine/chemokine mRNA transcripts within the neuroretina after immunization (Fig. 3E). Interestingly, expression of *Cxcl10* and *Cxcl11*, both of which have macrophage chemoattractant roles and are linked to autoimmunity and neuroinflammation [42–44], was most markedly upregulated in Nlrp12^−/−^ retinas. Although somewhat surprisingly, we did not observe a significant difference in induction of other myeloid-associated chemokines such as *Ccl2*. This suggests alternative pathways for Nlrp12-mediated chemotaxis within the eye beyond the traditionally defined Ccl2-pathway, which may favor other chemokines such as Cxcl10 or Cxcl11. These results indicate that Nlrp12 exerts a suppressive function in BM-derived myeloid cells, albeit it may also function locally in regulation of retinal cytokine/chemokine responses as evidenced by the BM-chimera experiments.

### A contribution for Nlrp12 in immune tolerance within the eye

**Fig. 4:**
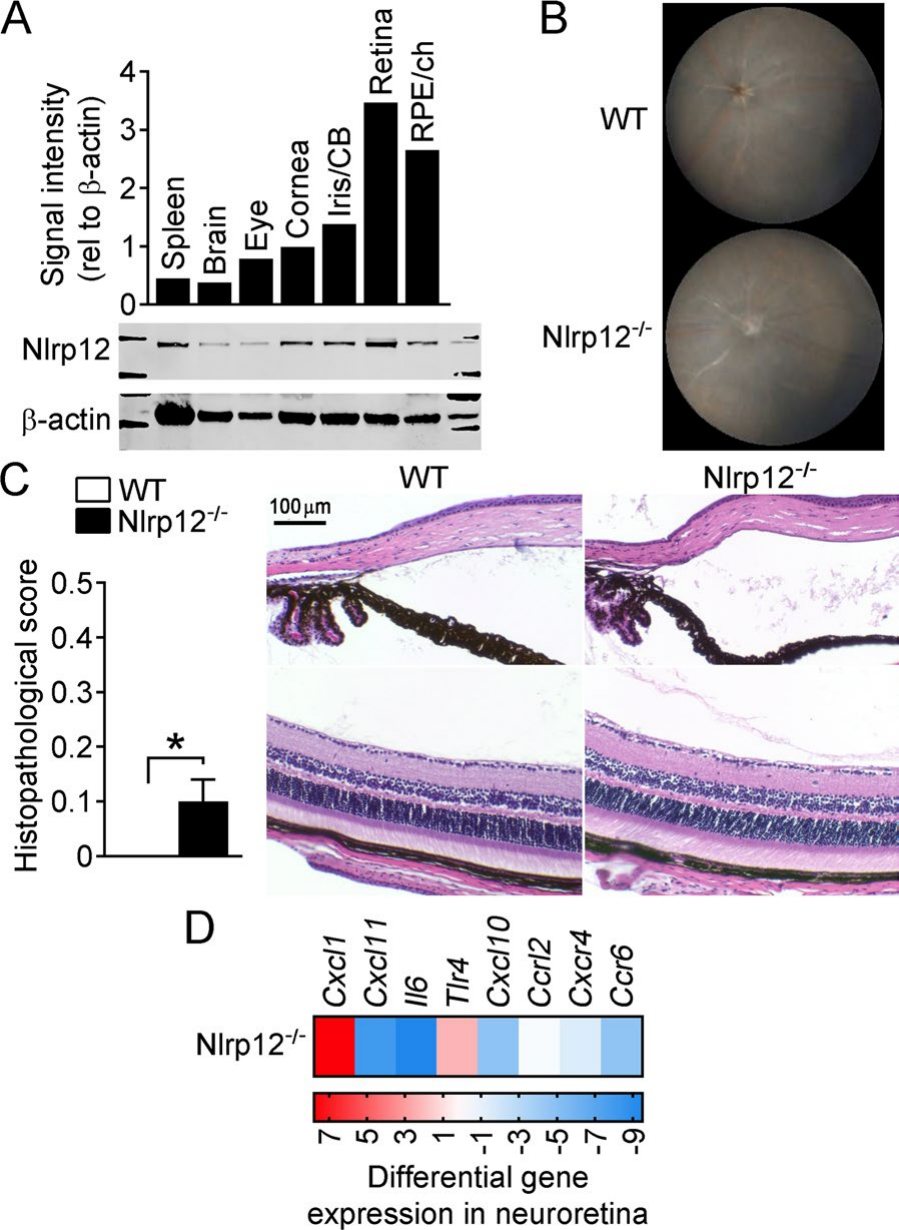
Contribution for Nlrp12 in immune tolerance within the eye. **A** Nlrp12 expression within distinct ocular tissues of naïve C57BL/6 J mice was assessed by Western blotting. Quantification by densitometry, as shown relative to β-actin expression (top), with representative immunoblot (bottom). **B**, **C** Ocular evaluation of adjuvant-injected WT and Nlrp12^−/−^ mice (from experiments presented in Fig. 1 above, *n* = 8 mice/group). **B** Representative fundoscopic images and **C** histopathology scores with representative images, *p < 0.05 by Mann–Whitney. **D** Expression levels of cytokine/chemokine genes were evaluated by multiplex RT-qPCR in neuroretinas dissected from adjuvant-immunized Nlrp12^−/−^ mice (14 day post-immunization). Colors in heatmap indicate the expression pattern of transcripts relative to adjuvant-injected WT (*n* = 5 retinas pooled/experimental group)

Closer examination of uveitis in BM-chimeric mice revealed that Nlrp12^−/−^ recipients transplanted with WT BM still developed significantly worse uveitis compared to WT controls (WT mice transplanted with WT BM) (Fig. 3D), thereby supporting a secondary, non-hematopoietic cellular role for Nlrp12. Since enhanced inflammatory transcription was also noted in Nlrp12^−/−^ retinas (Fig. 3E), we considered the potential contribution of resident ocular cells. When we surveyed Nlrp12 expression within distinct tissues of naïve eyes, we found Nlrp12 to be expressed in all tissues examined (Fig. 4A), though its expression was constitutively highest in the neuroretina and RPE/choroid, the tissues primarily affected by EAU. *Nlrp12* transcription across distinct ocular tissues was further confirmed (Additional file 1: Fig. S1).

In consideration of a potential retinal response controlled by Nlrp12, we more closely examined control WT vs. Nlrp12^−/−^ mice that were injected with adjuvant alone. Although not apparent by clinical examination (Fig. 4B), a subtle though statistically significant increased ocular response was detected with histopathology in adjuvant-injected Nlrp12^−/−^ mice, compared to WT mice (Fig. 4C). An increased amount of proteinaceous material, though not cells themselves, was detected in Nlrp12^−/−^ eyes, suggesting mild changes to the integrity of the vasculature and/or retinal tissue which were not apparent clinically. Intriguingly, at the molecular level, we also observed differential expression of transcripts in adjuvant-injected Nlrp12^−/−^ mice relative to the response in adjuvant-injected WT mice. In particular, upregulation of *Cxcl1* (Fig. 4D), which is important for recruitment of myeloid cells to the CNS, including the eye [45, 46], could permit subsequent ocular infiltration of other cells such as T cells. Together, these findings support an alternative function for Nlrp12 in the eye that may contribute to protection against EAU.

## Discussion

While NLRs play critical roles in health and disease, their functions have been poorly defined in uveitis. We used the EAU model, which has been instrumental in defining contributions of inflammatory mediators and T cell responses to pathogenesis of uveitis, to study Nlrp12 in integration of innate and T cell-mediated responses in ocular inflammation. We show, somewhat surprisingly, that Nlrp12 exerts an anti-inflammatory role in EAU to suppress uveitis. The exacerbated uveitis observed in Nlrp12^−/−^ mice was not attributed to inherent T cell dysfunction, but was due rather, to multi-cellular mechanisms involving BM-derived myeloid cellular responses as well as non-hematopoietic cell contributions. Nlrp12 was observed within ocular tissues, most notably within neuroretina, where it mediated induction of cytokines and chemokines.

A T cell-intrinsic mechanism for Nlrp12 is as yet to be resolved, as there have been conflicting findings in its role in EAE [31–33], a murine model for multiple sclerosis in which the CNS is the target organ. It is possible that differences in methodology, such as the amount of MOG_35–55_ peptide used for immunization (either 100 μg [33] or 200 μg [31, 32]), could affect patterns of cytokine production that might influence disease development. It is also conceivable that mice with genetically-engineered Nlrp12 alterations located in exon 2 [25] (used previously [33]) or exon 3 [24] (used in studies here and by others [32]) resulted in different animal phenotypes. Although Nlrp12^−/−^ mice have been reported to develop an attenuated form of classical EAE [33], we and others [32] found Nlrp12^−/−^ mice to be more susceptible to autoimmunity of the CNS. In any case, our data also did not support a T cell-intrinsic mechanism in protection against EAU.

Nlrp12 is predominately expressed in BM-derived cells of the myeloid lineage where it has been reported to negatively regulate inflammatory pathways [19, 47]. Hematopoietic-derived Nlrp12 has been reported to be important for host defense against *Klebsiella* infection, where bacterial clearance and neutrophil influx into the lung are dependent on Nlrp12 [48]. Our findings indicate that Nlrp12 is important in controlling responses of BM-derived myeloid cells such as neutrophils and macrophages, as a protective mechanism during EAU. These observations would be consistent with those of others using experimental models of colitis and colitis-associated colon cancer (CAC) [20, 25, 33, 49], where an inhibitory role for Nlrp12 is also involved in suppression of BM-derived macrophage recruitment and cytokine/chemokine expression.

In the eye, enhanced neutrophil and macrophage accumulation could, in turn, further potentiate IRBP-reactive T cell responses and more severe uveitis in Nlrp12^−/−^ mice. Thus, Nlrp12 may serve as an important immunomodulatory checkpoint in restricting autoimmune disease. This hypothesis would be substantiated by mutations (e.g. p.Arg284X) in *NLRP12* that result in impaired ability to suppress NF-κB activity [16] and inflammatory syndromes [50], and other missense mutations (p.Asp294Glu or Arg352Cys) that result in hyperactivation of the caspase-1 inflammasome [14]. Still, the precise molecular pathway by which Nlrp12 controls myeloid responses that protect against EAU remains to be determined in future studies. Historically, Nlrp12-mediated myeloid cellular responses may have been under-appreciated as a consequence of a missense mutation in *Nlrp12* in the C57BL/6J mouse strain, which recently came to light [51]. The Nlrp12 missense mutation was found to result in diminished neutrophil recruitment by macrophages. Our observations of heightened myeloid responses in Nlrp12^−/−^ mice and a BM-cellular mechanism, further lend support to the possibility of a Nlrp12-myeloid signaling axis as a dominant mechanism in uveitis.

The anti-inflammatory function of Nlrp12 has been previously documented in response to *Mycobacterium tuberculosis* [19, 23]. Accordingly, we detected molecular changes after peripheral injection of CFA-adjuvant alone (without IRBP), with retinal expression of *Cxcl1* and *Tlr4* increased in Nlrp12^−/−^ mice. This would support an additional function for Nlrp12 in retinal cells in protection against peripheral factors that may trigger breaches in the blood-ocular barrier and loss of immune privilege. Indeed, we not only showed Nlrp12 expression within the retina but also demonstrated a non-hematopoietic contribution of Nlrp12-mediated protection against EAU. Such combinatorial BM and non-BM derived roles for Nlrp12 have also been described in suppression of colon inflammation and cancer [20]. Collectively, our data support distinct tissue-specific functions for Nlrp12 involving suppression of retinal inflammation and BM-derived macrophage responses, which together protect against perpetuation of T cell-mediated uveitis.

The studies presented here are not without potential caveats. First, given the overlapping phenotypes between monocyte-derived macrophages and microglia, it has long been technically challenging to distinguish between their functions, particularly in scenarios of inflammation. However, it was recently discovered that monocyte-derived macrophages are of hematopoietic origin, whereas microglia are yolk-sac-derived and have a unique phenotypic signature that is conserved under steady-state and tissue injury conditions [52–54]. In the brain, microglia express Nlrp12, which functions to suppress production of reactive oxygen species and cytokines [32]. Networks of microglia also reside within the uveal tract and retina [41, 55], where they not only help maintain ocular homeostasis but also initiate inflammation in EAU [56]. Given the anatomical and developmental connection between the neuroretina and brain, it would be resonable to expect that retinal microglia also express Nlrp12. Based on our BM chimera studies and the markers used to phenotype the cell infiltrate in uveitic eyes of Nlrp12^−/−^ mice (CD45^high^CD11c^lo^MHCII^+^F4/80^+^), we have strong indication for a BM-monocyte-derived macrophage response rather than a microglia response. Examination of the microglia population by flow cytometry based on them being defined as CD45^lo^CD11b^lo^CD11c^lo^, did not show any difference between WT and Nlrp12^−/−^ (both being < 5% of the cells). However, we cannot exclude how their activation and/or function may be controlled by Nlrp12, as has been reported in purified microglia cultures from the brain [32]. This is notable since the retinal environment itself can also have potent influence over characteristics of infiltrated myeloid cells that render them to resemble endogenous microglia [57]. Thus, future investigation of how Nlrp12 controls microglia function would be of interest. Second, Nlrp12 expression was also found to be particularly high in the RPE/choroid complex. The RPE is known to play an important role in suppression of macrophage activation within the eye and protection against EAU [58]. Thus, our studies did not consider any putative mechanism of Nlrp12 that might be imparted by the RPE. Third, given several differences of cytokine/chemokine transcript regulation (e.g. of *Cxcl1* and *Cxcl10*) using RT-qPCR, it would be of interest to confirm these findings at the protein level, as well as future mechanistic studies to explore Nlrp12-mediated protection against uveitis. Last, Nlrp12 has recently been recognized to be involved in regulation of gastrointestinal commensal organisms that promote intestinal homeostasis [49, 59]. Nlrp12-deficient mice have increased susceptibility to colitis [20, 25] which can be partially mitigated with antibiotic treatment [49]. There is increasing evidence that disruption of commensal microflora (dysbiosis) is also connected to predisposition to ocular inflammation [60, 61]. While we found the mechanism for Nlrp12 protection against autoimmune uveitis to be predominantly of hematopoietic over non-hematopoietic origin (such as would be for the gastrointestinal tract), the extent to which Nlrp12 control of microbiota and/or intestinal cells contributes to protection against EAU remains to be fully determined.

## Conclusions

In summary, these findings uncover a novel role for Nlrp12 as a key genetic determinant of uveitis susceptibility, and the cellular mechanism by which Nlrp12 protects against uveitis could be targeted as an alternative approach for treatment of ocular autoimmunity.

## Supplementary Information

**Additional file 1: Figure S1.** Quantitative real-time PCR was used to evaluate mRNA expression of *Nlrp12* in distinct ocular tissues of naïve C57BL/6J mice. Data are shown as fold *Nlrp12* expression relative to that of spleen and are mean + SEM of 3 independent experiments (each sample contained RNA pooled from eyes of 6 mice/group).

## Data Availability

The datasets used and/or analyzed during the current study are available from the corresponding author on reasonable request.

## Abbreviations

NLR: Nod-like receptor
CNS: Central nervous system
CFA: Complete Freund’s Adjuvant
BM: Bone marrow
ELISA: Enzyme-linked immunosorbent assay
EAU: Experimental autoimmune uveitis
EAE: Experimental autoimmune encephalomyelitis
MS: Multiple sclerosis
IRBP: Interphotoreceptor retinoid-binding protein
TCR: T cell receptor
TEFI: Topical endoscopic fundus imaging
H&E: Hematoxylin and eosin
