# Supplementary material for: The innate immune receptor Nlrp12 suppresses autoimmunity to the retina
Source: J Neuroinflammation. 2022 Mar 21;19:69. doi: 10.1186/s12974-022-02425-x (PMC8939070; doi:10.1186/s12974-022-02425-x)
Supplement: Supplementary file 1 — Additional file 1: Figure S1. Quantitative real-time PCR was used to evaluate mRNA expression of Nlrp12 in distinct ocular tissues of naïve C57BL/6J mice. Data are shown as fold Nlrp12 expression relative to that of spleen and are mean + SEM of 3 independent experiments (each sample contained RNA pooled from eyes of 6 mice/group). [file 12974_2022_2425_MOESM1_ESM.docx]

**
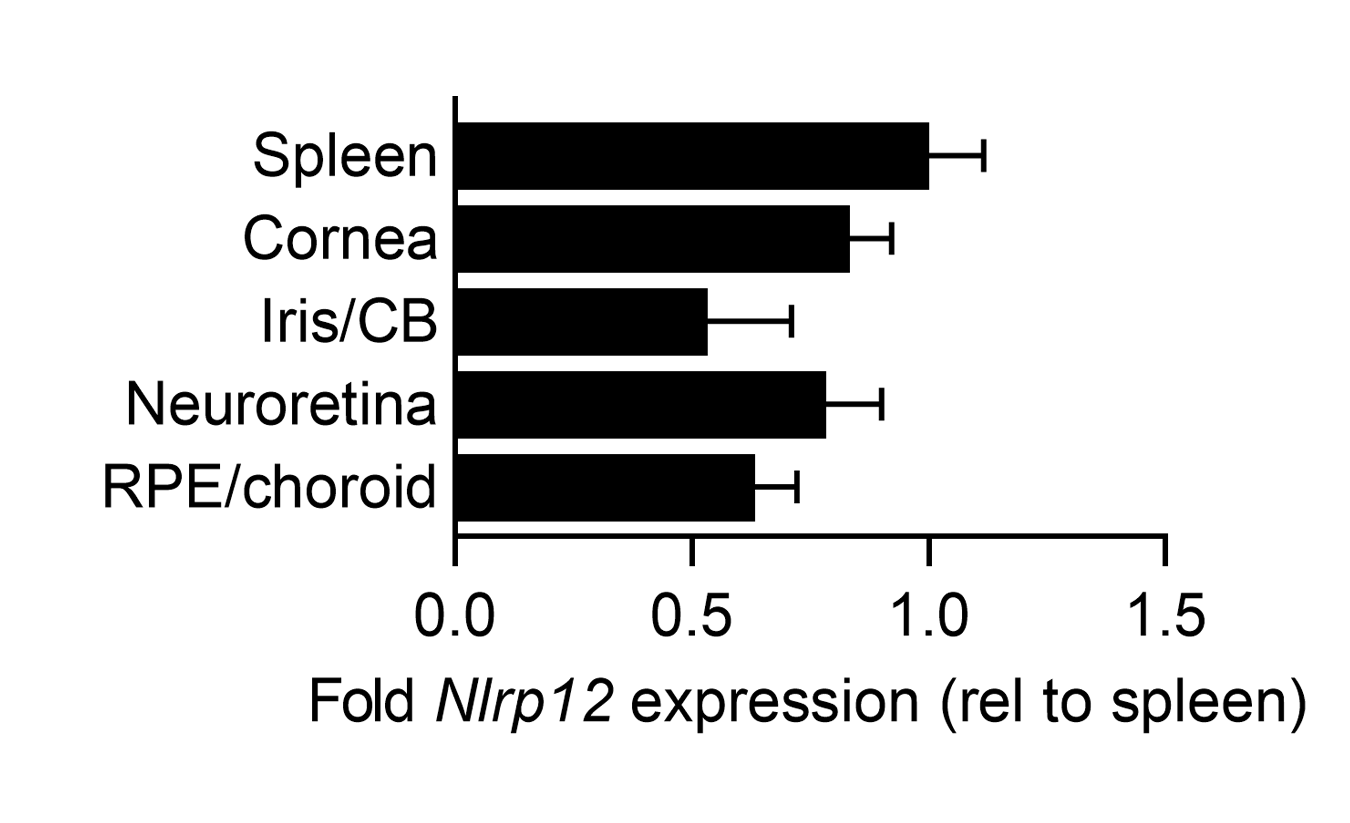
**

**Figure S1.** Quantitative real-time PCR was used to evaluate mRNA expression of *Nlrp12* in distinct ocular tissues of naïve C57BL/6J mice. Data are shown as fold *Nlrp12* expression relative to that of spleen and are mean + SEM of 3 independent experiments (each sample contained RNA pooled from eyes of 6 mice/group).
